# Electrochemical profiling of natural furanocoumarins: DNA interaction dynamics of oxypeucedanin and prantschimgin

**DOI:** 10.5599/admet.2199

**Published:** 2024-03-07

**Authors:** Hüseyin Oğuzhan Kaya, Gokay Albayrak, Hasan Isbilir, Fatma Kurul, Sura Baykan, Yeni Wahyuni Hartati, Seda Nur Topkaya

**Affiliations:** 1 Department of Analytical Chemistry, Faculty of Pharmacy, Izmir Katip Celebi University, Izmir, Türkiye; 2 Department of Pharmaceutical Botany, Faculty of Pharmacy, Izmir Katip Celebi University, Izmir, Türkiye; 3 Department of Nanoscience and Nanotechnology, Faculty of Science, Izmir Katip Celebi University, Izmir, Türkiye; 4 Izmir International Biomedicine and Genome Institute, Dokuz Eylul University, Izmir, Türkiye; 5 Department of Pharmaceutical Botany, Faculty of Pharmacy, Ege University, Izmir, Türkiye; 6 Department of Chemistry, Faculty of Mathematics and Natural Sciences, Universitas Padjadjaran, Indonesia

**Keywords:** Natural compounds, pencil graphite electrode, drug candidate, coumarins, electrochemical detection

## Abstract

**Background and purpose:**

In this study, we present an electrochemical sensor for the detection of oxypeucedanin (Oxyp) and prantschimgin (Pra), two natural furanocoumarin derivatives. The determination of the effects of these molecules on DNA is important to be potential drug candidates. Our research focused on exploring the electrochemical behaviour of these compounds and their interaction with DNA.

**Experimental approach:**

The electrochemical properties of Oxyp and Pra were systematically analyzed by evaluating their oxidation currents. Changes in the oxidation currents and peak potentials of guanine bases were monitored before and after interaction in the solution phase and at the electrode surface.

**Key results:**

The limit of detection (LOD) and limit of quantitation (LOQ) for Oxyp were determined to be 1.3 and 4.3 μg/mL, respectively. For Pra, the LOD and LOQ were found to be 20 and 68 μg/mL, respectively. Stability studies demonstrated that the Oxyp solution retained its oxidation capacity for over a month, whereas the Pra solution retained its oxidation capacity for nearly 120 min. Our findings suggest that Oxyp interacts with dsDNA, potentially through electrostatic interactions, showing promise as a potential drug candidate targeting DNA. On the other hand, the interaction of Pra with dsDNA requires further exploration to fully understand its mode of action.

**Conclusion:**

The electrochemical sensor developed in this study provides a reliable and efficient method for detecting and analysing the interaction of these natural compounds with dsDNA. Our research contributes to advancing the understanding of the interaction between natural furanocoumarins and dsDNA, laying the groundwork for the design and development of novel and effective DNA-targeted drugs.

## Introduction

Natural compounds are predominantly considered potential drug candidates or lead compounds for novel drug exploration in recent years [1]. This class of compounds includes coumarins, a group of secondary metabolites synthesized in plants through the metabolic pathway known as the shikimate pathway. They are divided into subunits based on their structural categories, such as simple coumarins, furanocoumarins, pyranocoumarins, 4-phenylcoumarins, and benzocoumarins [2]. Coumarins have a lactone ring formed structurally by benzene fused to an α-pyrone ring. The conjugated system in this structure possesses numerous electrons and exhibits good charge-transport characteristics. Some coumarins serve as additives in cosmetic products and aroma enhancers in tobacco and beverages [3]. They have been reported to possess anti-oxidant, anticancer, anti-microbial, analgesic, anti-diabetic, anti-inflammatory, and neuroprotective properties [4].

Two natural furanocoumarins, oxypeucedanin (Oxyp) and prantschimgin (Pra), have been identified in various plant species, such as *Angelica, Prangos, Ferulago*, and *Citrus* [1]. They have been extensively studied for their diverse biological activities, including anti-proliferative, cytotoxic, antispasmodic, anti-inflammatory, anti-influenza, and anti-allergic effects. Oxyp is often synthesized by plants as a part of their defense mechanisms or as secondary metabolites. While these compounds share some similarities, there are also distinct differences that set them apart. For instance, they both originate from the same biosynthetic pathway, the shikimate pathway, and possess a furanocoumarin skeleton. However, they are differentiated by their substitutes. Oxyp has 4-((3,3-dimethyloxiranyl)methoxy) side chain, while Pra has a 2-propan-2-yl 3-methylbut-2-enoate substitute and lacks a double bond in the furan ring. Oxyp has been extensively studied for its antioxidant, anti-inflammatory, and anticancer properties [2]. On the other hand, Pra has been investigated for its anti-bacterial, anti-viral, and anti-fungal activities [3]. The pharmacokinetic properties of Oxyp and Pra have not been thoroughly studied. Further research is necessary to elucidate the pharmacokinetic profiles of these compounds, contributing to a better understanding of their efficacy and safety in various therapeutic applications. Moreover, comprehensive research is needed to understand and compare the similarities and differences between Oxyp and Pra.

These compounds are isolated and purified from plant extracts using chromatographic techniques [4]. Other analytical techniques, such as nuclear magnetic resonance (NMR) [5], mass spectrometry (MS) [6], infrared (IR) spectroscopy, and ultraviolet-visible (UV-Vis) spectroscopy, [7] are also commonly employed for the identification and characterization of these compounds. In one study, UPLC/MS/MS was employed to determine Oxyp, one of the three major furanocoumarins in *Angelica dahurica* roots. The limit of quantification (LOQ) for Oxyp was 1 ng/mL [8]. In another study, the detection of Oxyp was carried out using the ^1^H-qNMR method, and the limit of detection (LOD) for the compound was found to be 0.149 mg/mL [9].

In another study, HPLC-DAD and HPLC-MS/MS, which combine a diode arrangement detector with mass spectrometry, were used to detect Oxyp. The lower LOD was found to be 0.1 μg/mL [10]. Previously, Pra was detected in *Ferulago* species by HPLC-DAD. LOD and LOQ for Pra were 0.264 and 0.879 μg/mL, respectively [3]. In another study with *Ferulago* species, two coumarins, felamedin and Pra were analyzed using HPLC. LOD and LOQ for Pra were determined as 0.0381 and 0.0177 mg/mL, respectively [11]. While these methods are commonly used for detecting Oxyp and Pra, they have some drawbacks, such as requiring burdensome pretreatment technology, large amounts of pure solvents, being time-consuming and labor-intensive, and often involving expensive and complex processes. On the other hand, electrochemical methods are advantageous as they overcome these disadvantages by providing a fast and reliable response, simple procedures, cost-effectiveness, low analysis time, and high sensitivity and selectivity [12]. Considering these advantages, electrochemical methods have gained prominence over the years in various fields for the detection of numerous organic substances, drugs, and various analytes in biological samples.

This study represents the first investigation of the electrochemical properties of Oxyp and Pra and their potential effects on double-stranded DNA (dsDNA). Initially, we explored the redox properties of Oxyp and Pra, followed by investigating the changes in oxidation currents and peak potentials of the guanine bases in dsDNA before and after interaction with these compounds. To optimize analytical signals, we carefully adjusted various experimental parameters, including concentration, pH, scan rate, and immobilization time. Furthermore, we assessed the stability of Oxyp and Pra at 4˚C. Notably, we successfully differentiated the signals of Oxyp and Pra in the same voltammogram, a distinctive feature of our study. Additionally, we utilized the SwissADME web tool to predict the in silico physicochemical properties, drug-likeness, and lead-likeness profiles of Oxyp and Pra, providing valuable insights for drug discovery and development. Furthermore, we calculated the toxicity values of these molecules with respect to their effect on DNA. The originality of this research lies in the comprehensive investigation of the electrochemical properties of Oxyp and Pra and their interactions with dsDNA, offering a novel perspective for drug research and pharmaceutical endeavours.

## Experimental

### Apparatus

Differential pulse voltammetry (DPV) and cyclic voltammetry (CV) measurements were conducted using an AUTOLAB potentiostat/galvanostat/impedance analyzer. The AUTOLAB was connected to a personal computer via a USB cable and operated using Nova software. The experimental setup consisted of a three-electrode system, with the working electrode being a pencil graphite electrode (PGE), a reference electrode made of Ag/AgCl, and a counter electrode composed of a platinum wire. The graphite lead was held in a Rotring T 0.5 mm pencil (Rotring, Germany). Pencil leads of HB grade, measuring 60 mm in length and 0.5 mm in diameter, were purchased from a local bookstore for use in the experiment. Column chromatography was carried out using silica gel 60 (70 to 230 mesh, Merck, Germany) and Lichroprep RP 18 (25 to 40 μm, Merck, Germany). A Varian Oxford AS400 (1H:400 MHz and 13C:100 MHz) apparatus was utilized to generate 1D NMR spectra. Mass spectra were obtained using a MALDI-TOF-MS spectrometer.

### Chemicals

Ethanol absolute (99.9 %) and glacial acetic acid were purchased from Isolab Chemicals. Fish sperm double-stranded DNA (dsDNA) was purchased from Sigma-Aldrich (Germany), and all other chemicals were of high purity, obtained from Merck (Darmstadt, Germany), Tokyo Chemical Industry C. LTD. (Tokyo, Japan). All solvents used were of high analytical grade and were employed without further purification. Buffers, including 0.5 M acetate (ACB) at pH levels of 3.8, 4.8 and 5.6, 0.05 M phosphate (PBS) at pH 7.4, 0.1 M sodium borate (BBS) at pH 9.8, along with 0.02 M NaCl and 0.05 M Tris-EDTA (TE) buffer at pH 8.0, were utilized in the experiments.

### Oxypeucedanin and prantschimgin

**Figure 1.**
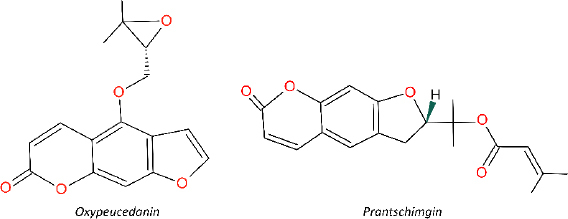
Chemical structure of oxypeucedanin and prantschimgin.

In our previous study, *Prangos uechtritzii* was obtained from the city of Konya of Türkiye [13]. The dried and powdered roots of the plant were sequentially extracted with n-hexane, chloroform, and methanol using an ultrasonic water bath for 24 h at room temperature. After filtration, the extracts were evaporated to dryness one by one at 40 °C under low pressure, yielding n-hexane (21 g), chloroform (19 g), and methanol (31 g) extracts. Column chromatography was used for fractionation and isolation studies. Following several chromatographic column studies, Oxyp (15 mg) and Pra (68 mg) were isolated and identified using 1D NMR and LC-MS in accordance with the literature [13]. The extracts and isolated molecules were subsequently lyophilized and stored at -20 °C. The chemical structures of Oxyp and Pra are shown in Figure 1.

## Experimental steps

### Activation of working electrodes

Before activation, PGEs were trimmed to a length of 3 cm. Subsequently, all PGEs were activated by applying a potential of +1.4 V for 30 seconds in an acetate buffer (ACB) at pH 4.8 to minimize background current. The PGEs, treated in this electrochemical process, were then used as working electrodes in the subsequent experiments.

### Electrochemical characterization of oxypeucedanin and prantschimgin

Before proceeding to the interaction studies of Oxyp and Pra with dsDNA, the electrochemical properties of both molecules were characterized.

Oxyp: 1 mg/mL stock solution of Oxyp was prepared with dimethyl formamide (DMF). Subsequently, it was diluted to a specific concentration with ACB (pH 4.8). Activated PGEs were immersed in this Oxyp solution and left for 1 hour for passive adsorption. At the end of the duration, PGEs were rinsed with ACB to remove unbound Oxyp from the electrode surface. Subsequently, DPV measurements were conducted. For CV measurements, 1 mg/mL stock solution of Oxyp was prepared with DMF. Subsequently, it was diluted to a specific concentration with ACB (pH: 4.8), placed into the measurement cell and measurements were conducted.

Pra: 1 mg/mL stock solution was prepared using DMF. Subsequently, it was diluted to a specific concentration with ACB (pH: 4.8). This Pra solution was placed into the measurement cell. DPV and CV measurements were conducted by immersing activated PGEs into this solution.

### Interaction

The interaction between Oxyp and Pra molecules with DNA was conducted both in the solution phase and on the electrode surface.

*At the electrode surface:* Stock solution of dsDNA (1 mg/mL) was prepared with TE buffer and, then diluted with ACB. The PGEs were immersed in the dsDNA solutions for 1 hour. After the immobilization, the electrodes were rinsed with ACB to remove unbound dsDNA.

In the subsequent step, these dsDNA-coated electrodes were immersed in Oxyp and Pra solutions at specific concentrations and left for 1 hour for interaction. At the end of the interaction, the electrodes were rinsed with ACB to remove the non-adhered Oxyp and Pra to the surface. Finally, measurements were conducted between 0.4 and 1.4 V using DPV.

*In the solution phase:* 1000 μg/mL of dsDNA and different concentrations of Oxyp and Pra were mixed in a 1:1 ratio and placed in a container suitable for stirring. Then, the PGEs were immersed in the solution and stirred at 650 rpm for 1 h at 37 °C. After the interaction of dsDNA and natural drug candidates, the PGEs were washed with ACB.

### Measurement

**Figure 2.**
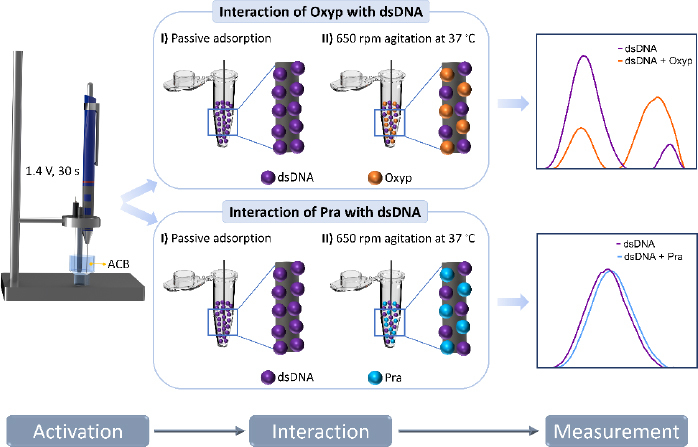
Schematic representation of experimental steps: activation of PGE; Interaction of dsDNA with Oxyp and Pra, and DPV measurements.

DPV measurements were performed in ACB at a scanning rate of 100 mV/s. CV measurements were performed at a scanning rate of 100 mV/s and a time interval of 0.05 s between each potential sweep. The experimental steps are illustrated in Figure 2.

### In silico physicochemical properties, drug-likeness, and lead-likeness prediction

The physicochemical properties, drug-likeness, and lead-likeness profiles of Oxyp and Pra were analyzed using the SwissADME web tool (http://www.swissadme.ch/) [14]. By inputting the chemical structures of the compounds, the tool provides valuable insights that aid in the evaluation of their pharmacokinetic profiles and potential as drug candidates. The tool predicts various parameters for the compounds, including molecular weight, numbers of hydrogen bond acceptor and donor topological polar surface area (TPSA), Lipinski's rule of five compliances, gastrointestinal (GI) absorption, blood-brain barrier (BBB) permeability, log *P* values, Ghose, Veber, Egan, and Muegge criteria. Additionally, it provides a bioavailability score, and identifies PAINS and lead-likeness criteria.

## Results and discussion

### Electrochemical properties of oxypeucedanin and prantschimgin

**Figure 3.**
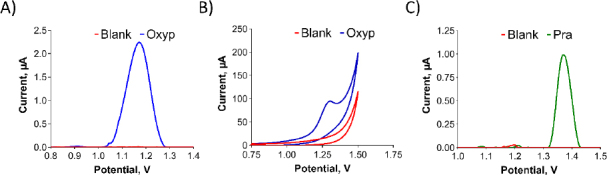
(A) Differential pulse voltammograms and (B) cyclic voltammograms of blank and Oxyp-coated electrodes. (DPV: +0.8 V to +1.4 V in ACB (pH 4.8) and *CV:* +0.65 V to +1.5 V at 100 mV/s scan rate with ACB (pH 4.8)). (C) DPV of ACB (pH 4.8) (blank) and Pra solution (Pra) with a scan rate of 100 mV/s and a range of +0.8 V to +1.4 V.

In Figure 3, we present the redox properties of the compounds. A stock solution of Oxyp (1 mg/mL) was prepared with DMF and diluted with ACB (pH:4.8). PGEs were activated and immersed in the Oxyp solution for 1 hour. The oxidation peak currents of Oxyp were subsequently measured with DPV in ACB (pH 4.8). Also, CV measurements were taken directly by dipping activated electrodes in Oxyp solution prepared with ACB (pH 4.8).

As shown in Figure 3A and Figure 3B, Oxyp exhibits a single oxidation peak potential at approximately +1.2 V. While attempts were made to investigate the reduction properties of Oxyp, a stable signal could not be obtained (data not shown). Consequently, we decided to focus on the oxidation currents of Oxyp for the rest of the study. A stock solution of Pra (1 mg/mL) was prepared with DMF and diluted with ACB (pH 4.8). Unlike Oxyp, various parameters (concentration, immobilization time, stirring, etc.) were tested, but Pra did not immobilize on the electrode surface by passive adsorption. Therefore, this Pra solution was added to the electrochemical measuring cell. Activated PGEs were immersed in these solutions and DPV measurements were performed. As shown in Figure 3C, Pra exhibits a single oxidation peak potential at approximately +1.4 V. Although attempts were made to investigate the reduction properties of Pra, a stable signal could not be obtained either (data not shown).

Determining the exact sites where molecules undergo oxidation is challenging; however, some conclusions can be made from the literature. It was observed that the oxidation signals of Oxyp and Pra were obtained at peak potentials close to each other. Upon examining these common structures in the literature, it is noted that the furan ring is electroactive and can be oxidized. The difference in the peak potentials of the oxidation signals is presumed to arise from variations in the substituent groups attached to the furan ring.

**Figure 4.**
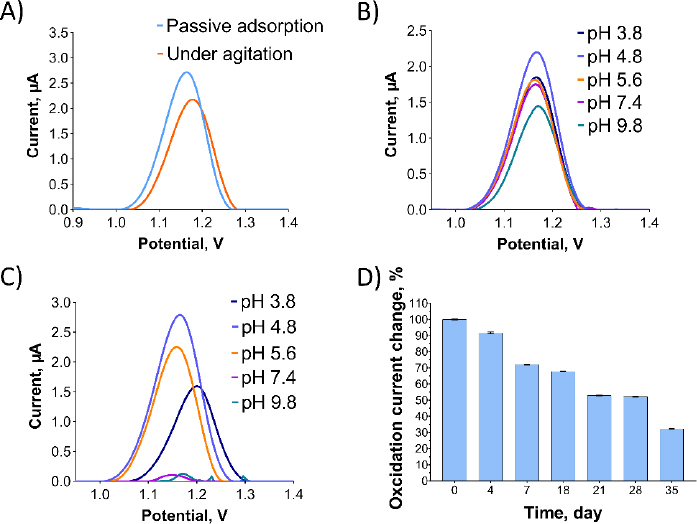
(A) DPV of oxidation currents of Oxyp with passive adsorption and agitating methods, scanning from +0.8 V to +1.4 V at 100 mV/s. (B) DPV of Oxyp prepared with different buffer solutions. (C) DPV of oxidation currents of Oxyp measured in different supporting electrolytes. (D) changes of Oxyp oxidation currents over a 35-day period at 4 °C, indicating the stability of Oxyp solution.

In Figure 4, optimization studies were conducted for Oxyp. In Figure 4A, two distinct methods for immobilizing Oxyp onto the electrode surfaces were compared: passive adsorption, where activated electrodes were dipped into the Oxyp solution, and agitation, involving dipping the electrodes with shaking. Figure 4A demonstrates that passive adsorption led to higher oxidation currents of Oxyp compared to agitation. Consequently, passive adsorption was selected for the subsequent phases of the study.

The impact of buffer pH and supporting electrolytes on Oxyp oxidation currents was investigated. In Figure 4B, an Oxyp solution was prepared with different buffer solutions ranging from pH 3.8 to 9.8. The highest Oxyp oxidation currents were recorded at pH 4.8. Consequently, pH 4.8 was selected as the optimal solution for the preparation of Oxyp. Figure 4C illustrates the influence of the pH of the supporting electrolyte on the oxidation currents of Oxyp. As the pH increased after 4.8, the amplitude of Oxyp oxidation currents significantly decreased. Moreover, the Oxyp oxidation peak potentials shifted slightly to negative values, indicating the involvement of protons in the oxidation process [15]. Since the highest oxidation peak was achieved at pH 4.8, this value was chosen as the supporting electrolyte's pH for further investigations.

We also conducted a stability study to assess the maximum oxidation capacity of the Oxyp solution. The Oxyp solution was prepared on day 0 and then stored at 4°C. As depicted in Figure 4D, the oxidation currents of Oxyp decreased by approximately 32 % on the 18^th^ day compared to day 0, demonstrating the decent stability of Oxyp. On the 35^th^ day of storage, the oxidation capacity of Oxyp is about 32 % compared to day 0. This indicates that where it can be used as a potential drug, improvements should be considered to increase its stability.

**Figure 5.**
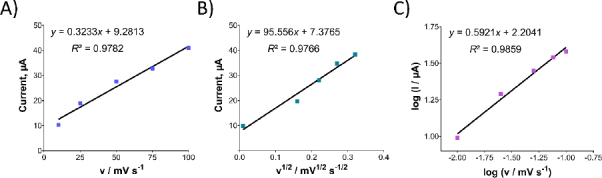
(A) Examination of the effect of scan rate on the peak of Oxyp’s oxidation current. (B) Analysis of the relationship between the scan rate root and peak current. (C) Investigation into the influence of scan rate on the logarithm of peak current.



After studying the impact of pH on Oxyp oxidation currents, we explored the effect of scan rate on the signal using CV in the range of 10-100 mV/s. As depicted in Figure 5A, Oxyp oxidation currents exhibited an increase with higher scan rates. The Equation 1 shows the relationship between the anodic peak current (I_pa_ and the scan rate (*v).*

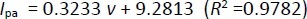

(1)


Figure 5B illustrates a linear relationship between the oxidation currents of Oxyp and the root of the scan rate, suggesting a diffusion-controlled process. This finding indicates that the electrochemical behavior of Oxyp is influenced by the rate at which species are transported to and from the electrode surface during the redox process [16]. The equation is as follows:



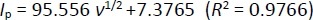

(2)


Figure 5C shows the relationship between the log of peak potential and the log of the scan rate. The equation is as follows:



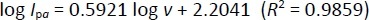

(3)


As the scan rate increased, the peak potential of Oxyp shifted towards more positive values, indicating an irreversible electrochemical process. This phenomenon occurs because the rate of the electrochemical reaction is governed by the diffusion rate of the reactants to the electrode surface. The diffusion process cannot keep up with the electrochemical reaction at higher scan rates, leading to an irreversible process [17].

This behavior aligns with findings from a previous study investigating the electrochemical detection of furanocoumarin derivatives imperatorin and umbelliferon. In that study, the oxidation peak potentials of imperatorin and umbelliferone also shifted towards more positive values with increasing scan rates, similar to our observations for Oxyp [18].

According to Equation 3, the slope value is close to the theoretical value, *e.g*., 0.5, indicating that the electrochemical process of Oxyp followed a diffusion-controlled mechanism. This result aligns with previous research, confirming that the rate of the electrochemical reaction is primarily governed by the diffusion of reactants to the electrode surface. The close correspondence between the experimental slope value and the theoretical value of 0.5 provides strong evidence supporting the diffusion-controlled nature of the Oxyp oxidation process, distinguishing it from an adsorption-controlled process [19].

**Figure 6.**
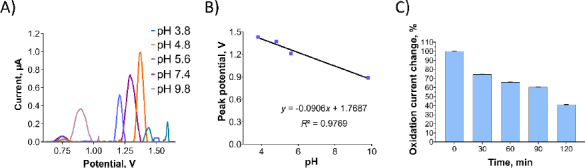
(A) DPV showing the oxidation currents of Pra prepared with various supporting electrolytes. (B) Plot illustrating the relationship between the oxidation peak currents of Pra and the pH of the supporting electrolytes. (C) Percentage changes in the oxidation currents of Oxyp over a period of 120 min.

In Figure 6A, the effect of the supporting electrolyte's pH on the oxidation currents of Pra was examined. As the pH increased, the oxidation current of the Pra exhibited a significant decrease. Additionally, the oxidation peak potentials of Pra shifted towards more negative values, indicating the involvement of protons in the oxidation process [15]. When the relationship between pH and oxidation peak potential is examined, along with the structure of the molecule, and considering the possibility of oxidation occurring at the furan ring, it is thought that during oxidation at the furan ring, 3 protons are involved per 2 electrons.

Among the tested pH values, the highest oxidation peak was observed at pH 4.8, which was selected for further investigations. The anodic peak potential (*E*_pa_) of Pra demonstrated a linear decrease as pH increased (Figure 6B). The relationship between *E*p_a_ and pH is presented by Equation (4):



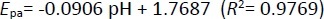

(4)


The slope value of 90.6 mV/pH from Equation (4) differs significantly from the optimal value of 59 mV/pH, indicating an unequal transfer of protons and electrons in the reaction. This discrepancy could arise from electrode process issues within the pH range, such as deprotonation or adsorption of oxidation products that might block the electrode surface. Despite testing various potential ranges and scan rates, Pra's oxidation currents were not observed in CV scan rate studies.

A stability study for Pra solution, similar to Oxyp, involved DPV measurements taken every 30 minutes for 120 minutes. In Figure 6C, the oxidation current of Pra decreased by approximately 60 % at 120 minutes compared to the 0 minute. This signifies low stability for Pra, highlighting the need for stability-enhancing formulations for potential drug use.

**Figure 7.**
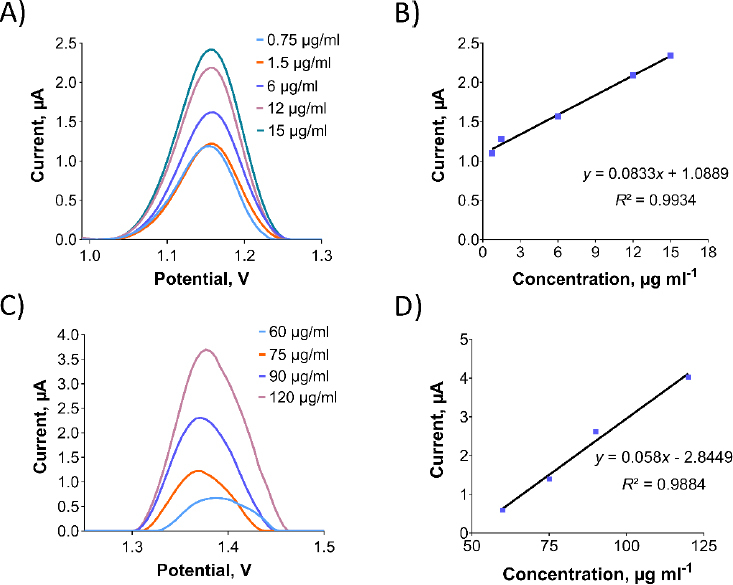
(A) DPV and (B) calibration plot for the different concentrations of Oxyp (0.75 to 15 μg/mL). (C) DPV and (D) calibration plot for the different concentrations of Pra (60 to 120 μg/mL).

After revealing the electrochemical characteristics of Oxyp and Pra, a concentration investigation was conducted. Various Oxyp concentrations ranging from 0.75 to 15 μg/mL were prepared, and their oxidation currents were measured with DPV (Figure 7A). The oxidation currents exhibited an increase with rising Oxyp concentration. Figure 7B shows that the oxidation currents of Oxyp exhibit a linear relationship between 0.75 and 15 μg/mL. The LOD was determined using the formula (3×standard deviation (SD*)*/(slope of calibration curve), and the LOQ was determined using the formula (10×SD)/(slope of calibration curve), where sd corresponds to the standard deviation calculated by regression analysis for a signal-to-noise ratio of 3. The study revealed that the LOD was 1.3 μg/mL, and the LOQ was 4.3 μg/mL for Oxyp.

Different Pra concentrations ranging from 60 to 120 μg/mL were prepared, and their oxidation currents were measured with DPV (Figure 7C). The oxidation currents showed an increase with higher Pra concentration. Figure 7D illustrates the linear relationship between the oxidation current of Pra and concentrations ranging from 60 to 120 μg/mL. LOD and LOQ for Pra were determined as 20 and 68 μg/mL, respectively.

**Figure 8.**
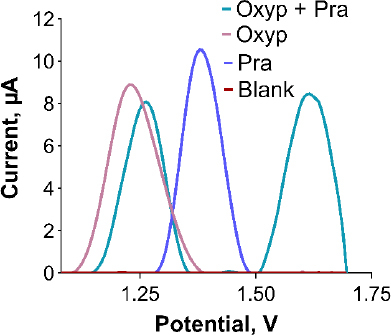
DPV obtained in different solutions (DPV: + 0.8 V to + 1.7 V at 100 mV/s scan rate with 0.5 s interval time).

Ensuring the sensor's selectivity for Oxyp and Pra is crucial. To assess this, a mixed solution containing both substances was prepared and individually distinguished. Initial solutions of 400 μg/mL Oxyp and Pra were prepared and mixed equally to form a 200 μg/mL solution of each, and separate 200 μg/mL solutions were also prepared. DPV measurements were conducted within the range of 0.8 to 1.7 V. Figure 8 shows the successful detection of distinct oxidation signals for Oxyp and Pra within the mixture, confirming the sensor's high selectivity. Minor reduction in oxidation peak height and more positive peak potentials suggest a potential interaction between Oxyp and Pra. When examining drug-DNA interaction studies in the literature, the shift of peak potential values towards a more positive value as a result of interaction indicates a possible intercalative interaction [20].

### The interaction of Oxyp with dsDNA

The assessment of the interaction potency between potential drug molecules and DNA constitutes a crucial aspect of pharmaceutical investigations. In our study, we tracked the oxidation currents associated with guanine and adenine bases in the solution phase and at the electrode interface before and after interaction with Oxyp and Pra. For the solution-phase interaction exploration, we mixed 1 mg/mL of dsDNA with varied concentrations of Oxyp solution, maintaining a 1:1 ratio. Consequently, this yielded a final solution containing 500 μg/mL of dsDNA, alongside the targeted Oxyp concentration. Following this, the electrodes were immersed in 100 μL of the interaction solution, and the tubes were placed onto a thermal shaker for 1 hour. Upon conclusion of this incubation period, we proceeded to quantify the oxidation currents of the guanine and adenine bases utilizing DPV, both in the presence and absence of Oxyp.

**Figure 9.**
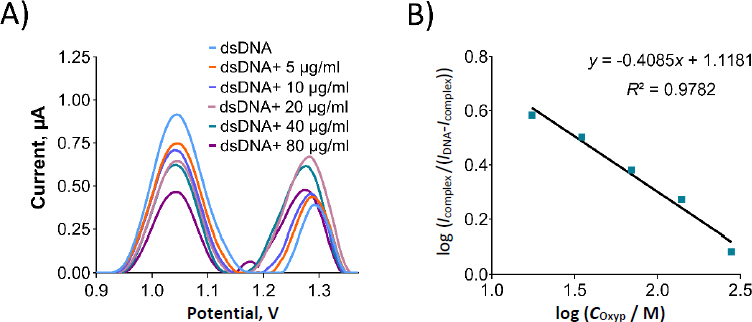
(A) DPV from 0.9 to 1.4 V at 100 mV/s of scan rate for dsDNA before and after interaction with Oxyp in solution phase. (B) Graph showing the linear relationship of log (*I*_complex_ /(*I*_DNA_-*I*_complex_)) *vs.* log *C*_Oxyp_ in the range of 17.47 to 279.45 μM.

We prepared Oxyp solutions with varying concentrations from 5 to 80 μg/mL (equivalent to 17.5 to 279.5 μM), which were subsequently combined with dsDNA in the solution phase. The oxidation currents corresponding to guanine were then quantified using DPV in both the presence and absence of Oxyp. As depicted in Figure 9, the oxidation currents of guanine exhibited a linear decrease as the concentration of Oxyp increased. The most significant decrease in the guanine oxidation current was observed at an Oxyp concentration of 80 μg/mL (279.5 μM). Consequently, this concentration was chosen for subsequent experiments.

**Figure 10.**
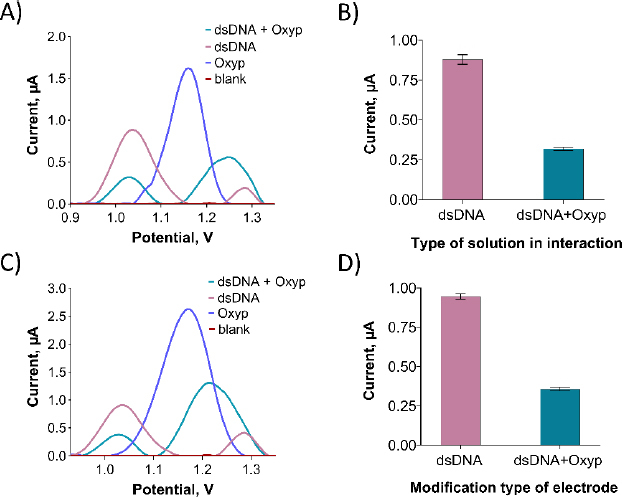
(A) DPV of Oxyp and dsDNA before and after interaction in the solution phase. (B) Histograms depicting the average oxidation current values of the guanine base before and after interaction with Oxyp in the solution phase. (C) DPV of Oxyp and dsDNA before and after interaction at the electrode surface. (D) Histograms illustrating the average oxidation current values of the guanine base before and after interaction with Oxyp at the electrode surface.

In Figure 10A, an oxidation signal was detected at approximately +1.22 V following the interaction between Oxyp and dsDNA in the solution phase. This signal was positioned between the oxidation signal of adenine at around +1.25 V and the Oxyp oxidation signal at approximately +1.20 V. We surmise that these two oxidation signals (adenine and Oxyp) converged post-interaction, leading us to exclude the adenine oxidation signal from our interaction assessment.

As depicted in Figure 10B, the oxidation peaks current of guanine exhibited a significant reduction following the interaction. Specifically, the guanine oxidation peak current experienced a decrease of 63.6 % after the interaction. Prior to the interaction, the guanine oxidation peak potential resided around +1.04 V, and following the interaction, it shifted slightly in the negative direction to approximately +1.02 V. This observation aligns with existing literature, suggesting an electrostatic nature of the interaction between Oxyp and dsDNA [21]. For electrode surface interaction, we prepared a 500 μg/mL dsDNA solution in TE and ACB. PGEs were immersed in this solution for 30 minutes, rinsed with ACB three times, and then dipped in 80 μg/mL Oxyp solution for 1 hour, followed by another ACB rinse.

As depicted in Figure 10C, following the interaction of Oxyp with dsDNA at the electrode surface, an oxidation signal at approximately +1.22 V was noted, positioned between the adenine oxidation signal at around +1.25 V and the Oxyp oxidation signal at about +1.20 V. Similar to the interaction in the solution phase, it is presumed that these two oxidation signals (adenine and Oxyp) merged after the interaction. In Figure 10D, a notable reduction in the oxidation peak current of guanine is evident following the interaction at the electrode surface. Specifically, the guanine oxidation peak current displayed a reduction of 59 % post-interaction.

By comparing the percentage reduction in the guanine oxidation signal due to interaction in both the solution phase and at the electrode surface, it is apparent that the interaction of Oxyp and dsDNA is more pronounced in the solution phase, albeit with a slight disparity at the electrode surface. Similar to the interaction studies in the solution phase, the guanine oxidation peak potential, initially situated at approximately +1.04 V before interaction, shifts slightly more negatively to around +1.02 V after interaction. This outcome provides further affirmation of an electrostatic mode of interaction.

### The interaction of Pra with dsDNA

To assess the interaction between dsDNA and Pra in the solution phase, we mixed 1 mg/mL of dsDNA with varying concentrations of Pra solution at a 1:1 ratio. This yielded a final solution containing 500 μg/mL of dsDNA and the desired Pra concentration. Subsequently, 100 μL of this interaction solution was utilized, and the electrodes were immersed. The tubes were then subjected to a thermal shaker for 1 hour. Finally, the oxidation currents of guanine and adenine bases were measured with DPV both in the presence and absence of Pra.

**Figure 11.**
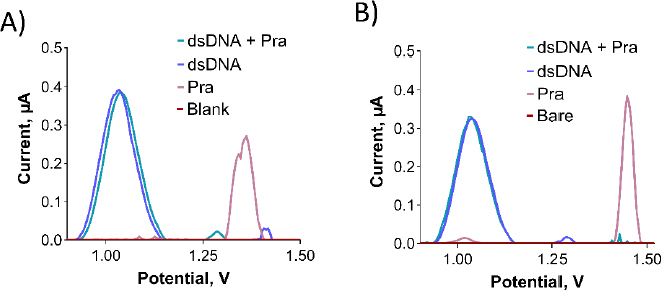
DPV of Oxyp and dsDNA before and after interaction (A) in the solution phase and (B) at the electrode surface.

As depicted in Figure 11A, no interaction was observed between dsDNA and Pra in the solution phase. This is evident from the nearly unchanged oxidation current of the guanine base following the interaction. Despite exploring various parameters, including different concentrations of dsDNA and Pra, as well as varying interaction times (data not shown), the outcome remained consistent.

To assess the interaction between dsDNA and Pra at the electrode surface, a 500 μg/mL of dsDNA solution was prepared using TE and ACB. PGEs were immersed in this solution for 30 minutes, followed by a single rinse with ACB. This procedure was repeated thrice. Subsequently, PGEs were immersed in the desired concentration of Pra solution for 1 hour and then rinsed with ACB. As illustrated in Figure 11B, no interaction was observed between dsDNA and Pra at the electrode surface, which is similar to the solution phase. This conclusion is based on the unchanged oxidation current of the guanine base after the interaction. Despite examining various parameters such as dsDNA and Pra concentrations, as well as interaction time (data not shown), the interaction did not manifest.

### The toxicity assessment

The toxicity value (*S* / %) of Oxyp on dsDNA was determined using Equation 5 after the interaction in the solution phase and at the electrode surface:



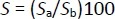

(5)


*S* / % Adenine peak current change

*S*_a_ / μA Magnitude of the guanine current upon interaction with Oxyp

*S*_b_ / μA Magnitude of the guanine current before interaction with Oxyp

Using Equation 5, we determined the *S* values to be 36 and 38 % for the solution phase and electrode surface, respectively. According to the literature, if the *S* value is greater than 85 %, the substance is considered non-toxic [22]. If the value ranges between 50 and 85 %, the molecule is moderately toxic to DNA, and if the value is below 50 %, it is considered toxic to DNA. Our results indicated that the Oxyp molecule could be toxic to DNA. Since there was no interaction between dsDNA and Pra, it was inferred that Pra did not exhibit toxicity towards dsDNA. Toxicity assessment could not be conducted due to the absence of signal change.

### In silico physicochemical properties, drug-likeness, and lead-likeness prediction

*In silico* evaluation of physicochemical, drug-likeness, and lead-likeness properties for Oxy and Pra is presented in Table 1.

**Table 1.** *In silico* physicochemical properties and drug-likeness prediction of Oxy and Pra.

Properties	Oxyp	Pra
MW	286.28 g/mol	328.40 g/mol
MLOGP	1.39	2.64
HBA	5	5
HBD	0	0
TPSA	0.6511 nm^2^	0.6574 nm^2^
XLOGP3	2.24	3.93
GI Absorption	High	High
BBB	Yes	Yes
Lipinski	Yes; 0 violation	Yes; 0 violation
Ghose	Yes	Yes
Veber	Yes	Yes
Egan	Yes	Yes
Muegge	Yes	Yes
Bioavailability Source	0.55	0.55
PAINS	0 alert	0 alert
Leadlikeness	Yes	Yes

The parameters were described by using Swiss ADME. MW: molecular weight; HBA: hydrogen-bond acceptor; HBD: hydrogen-bond donor; TPSA: topological polar surface area; MLOGP and XLOGP3: octanol-water distribution coefficients, BBB: the blood-brain barrier.

Lipinski, Ghose, Vever, Egan and Muegge rules are guidelines used in drug discovery to assess the drug-likeness of compounds based on factors such as molecular weight, lipophilicity, hydrogen bond donors and acceptors, polar surface area, and number of rotatable bonds, with the aim of predicting properties related to oral bioavailability, blood-brain barrier penetration, and overall pharmacokinetic behavior. PAINS is abbreviation for "Pan-Assay Interference Compounds." These are chemical substances commonly found in drug discovery screening tests that can lead to false positive results. The molecular weights of the compounds ranged from 286.28 to 328.40 g/mol. MLOGP and XLOGP3 values fell within the ranges of 1.39 to 2.64 and 2.24 to 3.93, respectively. The compounds exhibited 5 H-bond acceptors and 0 donors. TPSA values ranged from 0.6511 to 0.6574 nm^2^ (65.11 to 65.74 Å^2^). Both compounds displayed high gastrointestinal absorption and adhered to Lipinski’s rules based on these parameters [23]. Neither compound violated drug-likeness criteria from the literature as assessed by the web tool [24,25]. Furthermore, they showed no alerts against PAINS criteria [26]. Oxyp and Pra exhibited lead-likeness properties, indicating their potential as lead compounds for further synthesis studies.

## Conclusions

The electrochemical investigation has shed light on Oxyp and Pra behavior, unveiling potential avenues for DNA-targeting therapeutics. Evaluating their electrochemical properties and therapeutic potential on dsDNA provides a basis for drug discovery. This study pioneers Oxyp's and Pra's electrochemical detection and interaction with dsDNA. Oxyp's electrochemical properties were characterized, revealing an irreversible redox process at +1.2 V and a diffusion-controlled mechanism. LOD and LOQ were 1.3 and 4.3 μg/mL. We explored Oxyp's interaction with dsDNA in solution and at the electrode surface, tracking guanine's oxidation peak. Reduction in guanine's oxidation current post-interaction suggests Oxyp-dsDNA interaction. A shift towards more negative guanine oxidation potential indicates electrostatic interaction. Oxyp's toxicity towards dsDNA was assessed, revealing potential toxicity. Additionally, we investigated Pra's electrochemical behavior and dsDNA interaction. Unlike Oxyp, Pra did not exhibit significant interaction with dsDNA neither in solution nor at the electrode surface. The oxidation of guanine remained unchanged, indicating an absence of binding affinity. This selectivity reveals unique molecule-DNA interactions. Though Pra lacked interaction under our conditions, its therapeutic value merits further exploration. In conclusion, this study elucidates the electrochemical properties of Oxyp and Pra and their interactions with dsDNA. Oxyp demonstrates interaction potential with possible toxicity, while Pra exhibits limited interaction. These findings contribute to our understanding of bioactive molecule-DNA interactions, guiding innovative drug development and therapeutic strategies.
